# Involving end-users in the design of an audit and feedback intervention in the emergency department setting – a mixed methods study

**DOI:** 10.1186/s12913-019-4084-3

**Published:** 2019-04-29

**Authors:** Welmoed K. van Deen, Edward S. Cho, Kathryn Pustolski, Dennis Wixon, Shona Lamb, Thomas W. Valente, Michael Menchine

**Affiliations:** 1 Gehr Family Center for Health Systems Science, Department of Medicine, Keck School of Medicine, University of Southern California, 2020 Zonal Ave, IRD 318, Los Angeles, CA 90033 USA; 2 Cedars-Sinai Center for Outcomes Research and Education, Department of Medicine, Division for Health Services Research, Cedars-Sinai Medical Center, 116 N. Robertson Boulevard, PACT 801, Los Angeles, CA 90048 USA; 3 Keck School of Medicine, University of Southern California, 1975 Zonal Ave, Los Angeles, CA 90033 USA; 4 Interactive Media & Games Division, School of Cinematic Arts, University of Southern California, 900 West 34th Street, Los Angeles, CA 90089 USA; 5 Department of Preventive Medicine, Keck School of Medicine, University of Southern California, 2001 N Soto Street, Los Angeles, CA 90032 USA; 6 Department of Emergency Medicine, Keck School of Medicine, University of Southern California, 1200 N State Street, Room 1011, Los Angeles, CA 90033 USA

**Keywords:** Human centered design, Dashboard, Performance feedback, Audit and feedback, Emergency Department, Culture, Social network

## Abstract

**Background:**

Long length of stays (LOS) in emergency departments (ED) negatively affect quality of care. Ordering of inappropriate diagnostic tests contributes to long LOS and reduces quality of care. One strategy to change practice patterns is to use performance feedback dashboards for physicians. While this strategy has proven to be successful in multiple settings, the most effective ways to deliver such interventions remain unknown. Involving end-users in the process is likely important for a successful design and implementation of a performance dashboard within a specific workplace culture. This mixed methods study aimed to develop design requirements for an ED performance dashboard and to understand the role of culture and social networks in the adoption process.

**Methods:**

We performed 13 semi-structured interviews with attending physicians in different roles within a single public ED in the U.S. to get an in-depth understanding of physicians’ needs and concerns. Principles of human-centered design were used to translate these interviews into design requirements and to iteratively develop a front-end performance feedback dashboard. Pre- and post- surveys were used to evaluate the effect of the dashboard on physicians’ motivation and to measure their perception of the usefulness of the dashboard. Data on the ED culture and underlying social network were collected. Outcomes were compared between physicians involved in the human-centered design process, those with exposure to the design process through the ED social network, and those with limited exposure.

**Results:**

Key design requirements obtained from the interviews were ease of access, drilldown functionality, customization, and a visual data display including monthly time-trends and blinded peer-comparisons. Identified barriers included concerns about unintended consequences and the veracity of underlying data. The surveys revealed that the ED culture and social network are associated with reported usefulness of the dashboard. Additionally, physicians’ motivation was differentially affected by the dashboard based on their position in the social network.

**Conclusions:**

This study demonstrates the feasibility of designing a performance feedback dashboard using a human-centered design approach in the ED setting. Additionally, we show preliminary evidence that the culture and underlying social network are of key importance for successful adoption of a dashboard.

**Electronic supplementary material:**

The online version of this article (10.1186/s12913-019-4084-3) contains supplementary material, which is available to authorized users.

## Background

Emergency Departments (EDs) often face problems related to overcrowding and long waiting times [1] which leads to reduced quality and efficiency, as patients may decompensate or leave before being seen [2]. Long length of stay (LOS) in the ED is associated with higher seven-day mortality rates, more unnecessary hospital admissions [2, 3], and less patient-centered care [4]. Many factors likely contribute to long LOS, including patient volume and acuity, ED space and staffing [1, 2], and the number of diagnostic tests (e.g. lab and imaging tests) ordered [1, 5]. While many of these LOS factors are influenced by circumstances out of physicians’ control, the number of diagnostic tests ordered is directly influenced by the behavior of ED physicians. This is especially important as the number of lab and imaging tests ordered varies substantially across physicians and overutilization is a common quality concern [6–9], which delays the care delivery process and can cause harm to patients [10].

A variety of implementation strategies are used to change physicians’ practice patterns, ranging from educational methods to financial incentives and regulations [11]. Audit and feedback, the process of showing physicians performance feedback dashboards that compare their performance to their peers’, is a strategy that generally achieves small to moderate behavioral changes in healthcare settings [12]. Audit and feedback interventions work better when feedback is more intensive, [12] the reported outcomes are actionable [13, 14], and there is trust in the accuracy of the data [13]. Additionally, the perceived importance of the goal is important in achieving behavior change [15]. However, many questions remain about the most effective ways to deliver an audit and feedback intervention [16, 17].

The involvement of end-users in the design of the intervention is likely to improve its effectiveness, though this has yet to be formally evaluated in the ED setting [14]. Involvement of end-users mitigates the risk of creating an ineffective system that does not adequately meet users’ needs. This concept has long been understood in the field of human-centered design. Human-centered design is an approach that systematically incorporates end-user feedback throughout the design process [18]. This process helps to ensure that the design is functional, supports the end-user’s goals, and fits the organizational context [19]. Additionally, it ensures ease-of-use and usefulness of the technology, thereby engendering more positive attitudes towards the technology [20].

The implementation setting in which an audit and feedback intervention takes place also affects the desired behavior changes [12]. Organizational culture is thought to be a motivational force that shapes practitioners’ behaviors, attitudes, and thought-processes [21]. The underlying social networks, in turn, have been shown to impact culture and beliefs [22, 23]. Social networks also influence human behavior, as illustrated by evidence in the use of contraception [24] or attitudes towards smoking [25, 26]. The social network within the workplace has been shown to influence physician behavior as well [27, 28]. Additionally, the social network is integral to innovation diffusion, which demonstrates the role of interpersonal connections in the adoption of new technology [22, 29].

Here, we describe the development and exploratory evaluation of a performance feedback dashboard in the ED setting with the goal to reduce LOS. We employ a human-centered design approach to identify the most suitable measures to include on the dashboard and to ensure essential design requirements are met. We hypothesize that the systematic involvement of end-users in the design process will lead to a more appropriate intervention design and superior usability. This would improve physicians’ likelihood of using the performance feedback dashboard and increase their motivation to change practice patterns. Additionally, we hypothesize that both adoption and motivation are affected by the underlying culture and social network of the department.

## Methods

### Setting

This mixed methods study took place in the Los Angeles County + University of Southern California (LAC+USC) Medical Center ED. LAC+USC is a large public hospital owned and operated by the LAC Department of Health Services and is a major teaching hospital. The University of Southern California (USC) provides most of the attending services at the hospital. The ED is staffed by approximately 60 attending physicians and 70 ED residents. With over 100,000 patient visits per year, this ED is one of the largest in the country and serves predominantly uninsured and underinsured populations. The median LOS in 2016 was approximately 9 h for admitted patients and 6 h for discharged patients, which is well above the LOS observed in other EDs serving un- and underinsured populations [30]. Additionally, there was substantial variation between providers in LOS and utilization metrics; for example, the interquartile range (IQR) of the median LOS per provider was 6–9 h and the IQR of the % patients with a CT scan was 10–30%.

### Human-centered design approach

We employed a human-centered design approach to inform the development of a performance feedback dashboard for attending physicians. The goal of the performance dashboard was to reduce overall LOS by changing attending physicians’ practice patterns. Before developing the initial design, we performed a series of 60-min semi-structured interviews with ED leadership and ED attending physicians. Leadership interviews were performed to understand the strategic goals of the department and how a dashboard with specific performance measures could help attain those goals. Attending physician interviews were conducted to obtain end-user input on the measures to include and the design and functionality of the dashboard as well as to identify barriers to its implementation. The interview guides were developed specifically for this study (Additional file 1).

In total, two leadership and six attending physician interviews were performed to inform the initial design. The ED leaders were ED physicians whose primary responsibility was leadership but who also worked as attending physicians in this ED. We used a purposive sampling strategy to ensure a diverse representation of ED physicians. We included both male and female physicians with various levels of experience and different responsibilities (e.g. clinical, educational, academic, and administrative). Physicians employed by both USC and by the LA County Department of Health Services were included because incentive structures are different in the two settings. All participants were required to have worked in this ED for a minimum of 2 years to ensure they had a thorough understanding of the ED workflow. Twelve attending physicians were invited to participate, of which six completed interviews. Interviews were recorded and transcribed before analysis. Interviewees received a $25 gift card for their participation.

All transcripts were analyzed for comments related to relevant peer-comparison metrics, desired functionality, and perceived barriers. Based on the interview results, a guide was developed that specified design requirements for the performance dashboard. A team with experience in human-centered design developed (DW and KP) a front-end prototype in Axure RP PRO 8 (Axure Software Solutions, Inc., San Diego, CA). The design-team (DW, KP, ESC, WKvD) met on a bi-weekly basis to review the design and discuss appropriate solutions for design challenges. After the front-end prototype was developed using insights from the initial round of interviews, additional feedback was sought in a second round of 45-min interviews with five attending physicians, during which iterative improvements were made to the design.

### Survey

We conducted pre- and post-surveys (Additional file 2) 6 weeks apart to test whether the performance feedback dashboard (1) increases physicians’ motivation to make faster decisions about the patients’ management and (2) decreases physicians’ likelihood to order tests in a specific scenario; two outcomes that could lead to reductions in ED LOS. Screenshots of the prototype dashboard developed during the human-centered design process were displayed prior to the post-survey. Questions related to the culture and social network in the ED were included in the pre-survey, and feedback on the usability of the dashboard was obtained in the post-survey. Both surveys were hosted in Qualtrics (Provo, UT) and were electronically distributed to the 56 attending physicians that were active in the ED at the time the pre-survey was sent out. The pre-survey was sent out on January 1, 2018, with three reminders in the following month. The post-survey was sent out on March 13, 2018, with two reminders in the following month. Two OpenTable gift cards with $150 value were raffled among participants as an incentive to complete the pre-survey. In the post-survey, a $5 Starbucks gift card was offered to each participant as an incentive.

### Measures

The primary outcome was the effect of the performance feedback dashboard on physicians’ motivation to make a quick disposition decision (i.e. the moment the physician decides whether the patients will be admitted to the hospital or discharged from the ED). To measure motivation, we used a set of measures derived from Self-Determination Theory which posits that perceived value/usefulness, competence, and autonomy support are important factors for motivation [15]. We used the Value/Usefulness and Competence subscales of the Intrinsic Motivation Inventory and the six-question version of the Work Climate Questionnaire for Perceived Autonomy Support [31], which have been used and validated in a variety of settings including educational settings, sports psychology, and the workplace environment [32, 33]. All questions were rated on a seven-point Likert scale ranging from “Not at all true” to “Very true”. These questions were included in both the pre- and post-survey.

To estimate changes in physicians’ practice patterns, we focused on test-ordering, which is a known predictor of LOS and is directly influenced by physicians’ decisions [1, 5]. To assess physicians’ likelihood of ordering diagnostics tests, we included a vignette adapted from Kool et al. [34] in which the need for imaging is purposefully ambiguous. After reviewing the vignette, physicians were asked whether they would order diagnostic imaging, and if so, what type of diagnostic order. The vignette was included in both the pre- and post-survey.

To evaluate the design of the prototype performance dashboard in the post-survey, we assessed the perceived usefulness and ease of use based on the Technology Acceptance Model [20]. The Technology Acceptance Model has been used extensively to evaluate physician acceptance of health technology [35, 36]. Questions such as “I believe that the performance feedback tool is flexible to interact with” were scored on a seven-point Likert-scale ranging from “Disagree strongly” and “Agree strongly”. We also assessed physicians’ perception of the importance of the metrics included in the dashboard and physicians’ perceptions of their ability to affect these measures on the same seven-point Likert-scale. Lastly, we asked physicians a Net Promotor Score question, which is widely used in various industries to understand the quality of the product or service: *“How likely are you to recommend this performance feedback tool to your colleagues and peers to visualize and improve their performance as ED physicians?”* with an eleven-point response scale ranging from “Not likely at all” to “Extremely likely.” We also asked for suggestions to improve the tool and inquired about additional physician comments.

To assess the ED culture, we asked physicians about their perceptions of the social, technical, and environmental aspects of their workplace in the pre-survey. We used the first thirteen questions of the Safety Attitudes and Safety Climate Questionnaire, which has been extensively psychometrically validated [21, 37]. These thirteen questions specifically address the teamwork and safety climate. All questions were scored on a five-point Likert scale ranging from “Disagree strongly” to “Agree strongly”, with an N/A option for each question. To describe the underlying social network, we included the question “*Who do you discuss problems with at work?*” which returns social network data indicating specifically who communicates with whom in the organization [38]. Understanding this network is important if there are cultural barriers that limit the adoption of an intervention [39]. Up to seven names could be selected from a pre-specified list of ED provider-names, including those in leadership roles. See Additional file 2 for the complete surveys.

### Statistical analysis

To assess the importance of end-user involvement in the development process, respondents were categorized as (1) directly involved in the dashboard development (interviewees, co-author MM), (2) not directly involved in the development but ≥30% of their network was involved, or (3) not directly involved and < 30% of their network was involved. The social network question was used to create a model that assessed the level of exposure that each participant had to people who were involved in the development of the dashboard. Exposure was defined as the percentage of nominated people who were involved in the development [23].

Centrality measures for the social network analysis (in-degree, out-degree, closeness, and betweenness) were calculated using the igraph library [40]. Out-degree is the number of people someone selected in the network question; in-degree is the number of times a person was selected by someone else; closeness measures the average distance someone has to everyone else in the network; and betweenness is a measure of how often each person lies on the shortest paths connecting others [41]. People with high betweenness scores are thought to be key in the early adoption of innovation, people with a high in-degree are ‘popular’ figures in the network that are important for the innovation to spread to the early majority, and people with a high closeness score are thought to be important for an innovation to spread to a large amount of people [41].

Descriptive statistics were used to describe the sample and survey results. Pre- and post-survey data were compared using a Wilcoxon Ranked Sum test for continuous measures and the McNemar’s Test for categorical variables. The Kruskal-Wallis Test was used to compare change-scores between groups. A Spearman correlation coefficient was used to describe relations between continuous data. Rho correlations coefficients of > 0.7 were considered strong correlations, coefficients between 0.5–0.7 were considered moderate, and coefficients between 0.3 and 0.5 were considered weak. A *p*-value of < 0.05 was considered significant. Analyses were performed in SAS 9.4 (Cary, NC).

## Results

### Human-centered design approach - interviews

A total of eight semi-structured interviews were performed to guide the initial design of the performance feedback dashboard between May and August 2017 (Additional file 3: Table S1). Half of the interviewees were male, their median time since starting residency was 12 years (range 9–45), and their median length of employment in the current ED was 8 years (range 2–31). Two of the interviewees had primarily leadership roles, three primarily clinical responsibilities, and three primarily academic responsibilities. Two were employed by the LA County Department of Health and six by USC. The median duration of the interviews was 57 min (range 35–77). Thematic saturation was achieved after seven interviews; no new themes emerged in the eighth interview.

#### Dashboard content – metrics

Numerous metrics were discussed in the interviews, including the number of patients, their discharge disposition, and a variety of length of stay, utilization, and outcomes measures (Additional file 3: Table S2). Overall, the time between the moment a physician first sees the patient until the moment a disposition decision is made (i.e. decision to admit a patient to the hospital or discharge from the ED), was perceived to be the most relevant LOS metric. “*Provider to decision time. Yeah, I think that’s an important metric, I think that there’s probably some wiggle room in there. In general, I think that’s an important part of our job is to have a disposition decision within whatever time frame we deem to be appropriate*.” (Interview 7).

Additionally, test utilization, including CT scans, MR scans, and labs, was thought to be of interest, especially if compared to peer utilization. *“I think the number of yes [sic], I order 500 CT-scans in a year, and somebody else orders three, would be interesting. It would be interesting to know the false positives and the false [negatives].”* (Interview 5). Interviewees also expressed an interest to know the outcomes of their decisions, or what happened to patients after they left the ED: “*Bounce backs, maybe? Stuff that’s, like, more subjective, like, you know, your decisions on the patient. [ …*] *you admit someone to the floor and they die. Should they have gone to the ICU? Or you discharge them and they die. [ …*] *That’d be nice to know.*” (Interview 2).

#### Dashboard content – comparisons

Interviewees expressed the importance of comparing their data to others and of including historic trends. “*So ideally [the data] would be where you are, where you were the previous month, or the previous time last year. And then where the department is.*” (Interview 8). Ideally data would have to be aggregated on a weekly or monthly basis, as a day would not provide useful information because shifts vary day to day, while a year would be too long to remember. “*[ …*] *if you wait too long then it doesn’t make any sense to you because you’re like, ‘That happened in September of last year. I don’t care. I don’t know what happened then.’ If you do it every day, then they’re kind of like, ‘Well, how am I supposed to know what I’m supposed to change?’*” (Interview 2). Interviewees wanted to receive their data compared to their peers in a blinded fashion. Some expressed discomfort with others seeing their data: “*I’m personally um, scared you know, scared to be compared with others.*” (Interview 3). Others didn’t think it would be necessary to share the data openly to achieve change: “*We serve as our sort of biggest critics already and that for us to see it is enough to be like a blow to an ego where you can say, ‘Oh wow, I’m that slow compared to my colleagues?’* ” (Interview 7).

#### Dashboard functionality

Three key themes emerged in the discussions about desired functionality of the performance feedback dashboard. First, participants emphasized the need for the dashboard to be easily accessible to increase the likelihood that people would actually use the dashboard. “*If it’s like one more thing to do, I might not do it. It would have, it would have to be something that was like every time you logged in it popped up or something, or I would not look at it. Yeah. I’m not gonna go seek out that information.”* (Interview 4). On the other hand, physicians did want to be able to drill down the data further to know what happened during specific shifts or with specific patients. “*Ideally for people that have unscheduled returns, I would like to see why ... I would like their name, their medical record number so I could look them up and also then what happened to them on the return visit.”* (Interview 8). Lastly, interviewees expressed that they would like to see a certain level of customization to define metrics of interest for them: “a *good dashboard should give you the ability to, um, customize it, customize the view and the parameters that you look at for your, for your position at that time. Um, and you should be able to change that on the fly.*” (Interview 1).

#### Barriers and unintended consequences

A variety of concerns were brought up during the interviews. A performance dashboard might increase physician stress levels, dehumanize the patient experience, and potentially negatively affect the quality and safety of care: “*Yeah, if I’m seeing four patients an hour and I need to have a dispo [disposition decision] on each of them within an hour and a half or two hours or whatever the ED group tells you is the right metric then probably I’m cutting a lot of corners not having substance of conversations with the patient, not providing great patient care.*” (Interview 7). Additional concerns were related to physicians’ responsibility to teach the many residents in the ED, which could be negatively affected. “*A lot of times we give the residents a lot of leeway to think things through. And part of that is that we haven’t had to move on a very like super-fast time process. [ …*] *if you’re having time in the ED as something you’re thinking about more than necessarily clinical accuracy, it’s ... it is gonna change the way we practice with supervision.”* (Interview 4).

Data accuracy was a concern as well, given that electronic medical record (EMR) data might not always be accurate. *“All of your metrics are only as good as the people putting them in, and that’s always gonna be, you know, um, an issue with accuracy [ …*]. *”* (Interview 1). Interviewees also expressed the need to correct for the acuity of patients seen and the shift. *“Oh, if you work more night shifts you’re gonna see more of them [more challenging cases]. Probably have to control for shift in some way.”* (Interview 4). Additionally, concerns were expressed regarding gaming of the system and problems with attribution of patients to the right physician, especially in a teaching hospital with many residents. *“When they’re finally put in to a room, they very well may see a different resident and attending at that point. So, there may be three different [physicians involved]. And that’s not even counting change of shift.*” (Interview 8).

### Human centered design – initial design and iterative improvement

Based on the interviews, we designed an initial version of the performance feedback dashboard between August and November 2017, which was then iteratively improved in two additional design cycles based on additional end-user feedback from five physicians in November and December 2017 (Additional file 3: Table S1). The first major iteration was developed after three additional interviews; the second and last major iteration was developed after another two interviews that revealed only minor required changes (Table 1), after which the design process was finalized.

**Table 1:** Design decisions made during human-centered design

Interview observations	Design solution - initial design	Rationale	Iterative improvement based on follow-up interviews
Metrics			
a) Summary measures	n patients, n patients by area, n patients by shift, n patients by discharge disposition	To give physicians an overview of the patients they saw in a month. Show the area of care and shifts as it might affect case mix	
b) Length of stay metrics	Provider to decision time, overall LOS^a^ (median)	Overall LOS is most important for ED, but provider-decision time is easier to influence by provider	‘Provider to decision time’ changed to ‘Room to decision time’ as patients might see another provider in the waiting area (iteration 1)
c) Utilization of tests	CT, MR, US, lab utilization (%)	Can be affected by physician and is known to affect LOS	
d) Outcomes	72-h return rates, deaths (%), LOS after admission (median)	To address concerns about negative outcomes, return-rates and deaths were included. LOS after admission was included as a proxy measure of appropriateness of admissions	Removed deaths as an outcome as it is not feasible to reliably obtain data from EMR (iteration 1)
Comparisons			
a) Over time	Monthly intervals	Balance between too frequent reports with random variation and too infrequent where physicians don’t remember what happened	
b) To peers	Blinded ranking (e.g. ‘your ranking 46/60’, with outcomes of peers with better or worse numbers shown). Overall ED medians are shown on separate page	Blinded since all interviewed physicians agreed un-blinded was not desired/needed. A ranking showing neighboring peers was included to give physicians an attainable goal	Changed the ranking to interquartile range of peers instead, since the optimum rate is likely someplace in the middle, outliers in either direction can be a problem (iteration 1)
Functionality			
a) Ease of access	Monthly email summary with 3 measures that can be selected by ED leadership based on priorities	Easy to access	
b) Drilldown functionality	Option to access full dashboard through a link in monthly email	Drilldown functionality	Added tabs to drill down based on the type of shift (e.g. night) and assigned area. (iteration 1)
c) Customization	Physicians can select measures to show up on their own favorites page Leadership can select measures in monthly email	Customization options for individual physicians and leadership based on ED priorities	
Barriers			
a) Adverse consequences on quality of care	Inclusion of outcomes on dashboard	To avoid focus only on throughput and utilization measures, which might result in adverse consequences	
b) Conflicting teaching responsibilities	–	As this was not the goal of the dashboard, no measures related to teaching were included	
c) Data accuracy	–	Extensive validation of data is required	Included definition of the measures on the dashboard. Made section headers very clear (iteration 2)
d) Case-mix adjustment	Show total number of patients during different shifts and in different areas of care.	By showing these measures, physicians can put other measures in context.	Added tabs to drilldown by area of care and type of shift (iteration 1)

*Abbreviations*: *CT* computed tomography scan, *ED* emergency department, *EMR* electronic medical record, *n* number, *MR* magnetic resonance imaging, *US* ultrasound

^a^Overall LOS is only shown on the overall ED page, not on individual physician dashboards

**Fig. 1:**
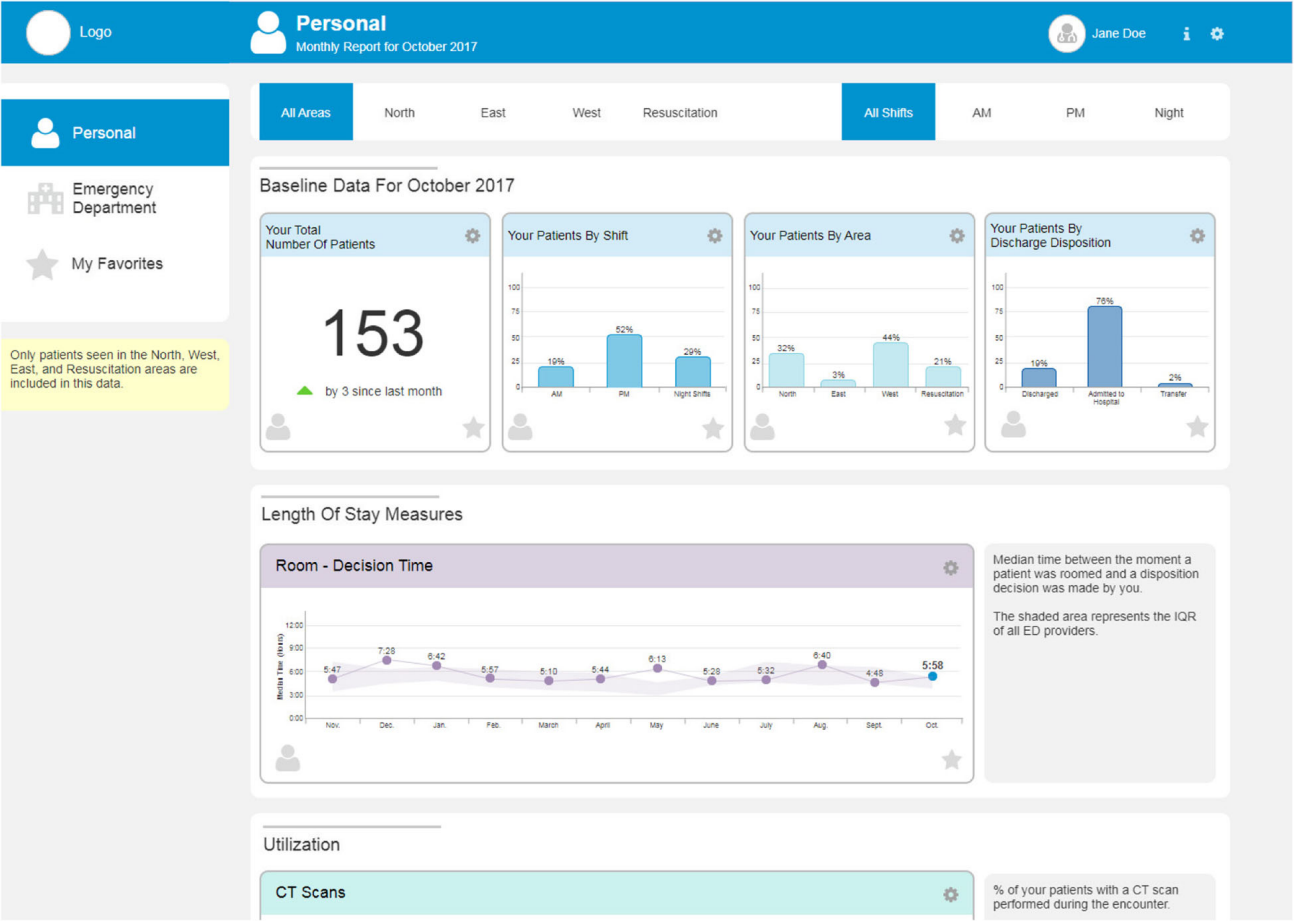
Top section of performance feedback dashboard. The top section of the designed prototype performance feedback dashboard. The name Jane Doe is a false name

The design incorporates a monthly summary email that includes a few selected measures to ensure the data is easy to access (Additional file 3: Figure S1). A link would be provided to an interactive performance dashboard with a variety of measures that can be accessed on demand (Additional file 3: Figure S2), to give physicians the opportunity to dive deeper into the data if desired. The interactive dashboard includes three pages: a “Personal” page showing a physician’s individual performance compared to their peers, an “Emergency Department” page showing the performance of the ED overall as a reference, and a “My Favorites” page that displays measures selected by individual physicians (Fig. 1). During the iterative interviews, tabs were added to the pages to allow physicians to drill down by the timing of shift and the area of care within the ED as this reflects the severity of the cases, thereby providing an opportunity to adjust for case-mix. Table 1 gives a complete overview of the design considerations in the human-centered design process and the iterative improvements made.

The selection of measures for the dashboard was informed by audit and feedback best practices [12–14] and on information obtained during interviews in accordance with a human-centered design approach [18]. We specifically ensured a selection of measures that are actionable to physicians as this is a known requirement for effective audit and feedback interventions [13, 14]. The primary goal of the dashboard was to reduce overall patient LOS. However, to increase actionability of the measure, we focused on the time that can be influenced directly by physicians. In initial versions, this was the time from first physician to the time the physician makes the disposition decision (i.e. decision to admit to hospital, observation, or discharge). After additional interviews, we modified this to the time from when the patient is roomed until the disposition decision is made, as this more accurately represents the period directly influenced by the attending physician.

Test utilization data, including the percentage of patients with a CT scan, MR scan, ultrasound, and lab test, were included because they affect LOS and are directly controlled by the physician. The number of requested consultations was not included as the data were not readily available in the EMR. Outcome data were included to address concerns about unintended consequences related to the quality of care if the focus was merely on LOS and test utilization. Initially, we included 72-h return rates and number of deaths. However, deaths were excluded from later versions in response to concerns about the reliability of EMR-derived death-data. Hospital LOS after admission was included as a proxy measure of appropriateness of admission, with the assumption that a short LOS might indicate that the patient did not need to be admitted. The total number of patients with the breakdown by timing of shift, area of care, and discharge disposition was included to provide more context about the patient population seen. During iterative rounds of improvements, we added detailed definitions of the measures used and clarified section headers to improve comprehension and trust in the dashboard.

It was decided to report data by monthly intervals to minimize the effect of day-to-day random variation, but to also ensure physicians still remember what happened during a given period. Comparison data were provided on the “Emergency Department” page showing summary measures for the entire ED. On initial designs, comparison data was shown on the “Personal” page as a ranking that included blinded peer data to provide physicians with achievable goals for improvement. However, further feedback made it clear that a ranking was not perceived as appropriate because the highest or lowest score does not necessarily indicate the best or the worst performance. Instead, it was decided to show the interquartile range of all physicians.

### Survey

In total, 19 attending physicians (34%) responded to both pre- and post-surveys. Forty-two physicians (75%) responded to the pre-survey which included the clinical vignette and measures for value/interest, perceived competence, autonomy support, teamwork climate, and safety climate. Twenty-one physicians (37.5%) responded to the post-survey which included a mockup of the performance feedback dashboard, the clinical vignette, measures for value/interest, perceived competence, autonomy support, and the perceived usefulness of the dashboard and the perceived ease of use (Additional file 3: Table S3). Of the 19 physicians that responded to both surveys, 5 had been involved in the development of the dashboard, 7 physicians had more than 30% of their direct network involved in the development, 5 physicians had less than 30% of their direct network involved, and 2 did not answer the network question. Physicians with less exposure to those involved in the development process tended to have less experience and physicians who were directly involved in the development tended to have a more central position in the ED social network. Although all groups selected a roughly equal number of people to ask for advice (out-degree), physicians who were involved in the development were selected more frequently by others (in-degree), and had numerically higher betweenness scores. Closeness scores were similar between groups (Table 2).

**Table 2:** Characteristics of survey respondents

	Involved	≥30% of network involved^a^	< 30% of network involved^a^	*p*-value
	(*n* = 5)	(*n* = 7)	(*n* = 5)	
Male gender – *n (%)*	3/5	5/7	4/5	1
Years experience – *median (IQR)*	10 (10–15)	10 (9–14)	4 (3–9)	0.15
Years in this ED – *median (IQR)*	10 (6–13)	7 (2–9)	2 (1–5)	0.29
Network centrality measures^a^				
- Out-degree – *median (IQR)*	6 (4–7)	5 (3–7)	6 (5–7)	0.88
- In-degree – *median (IQR)*	6 (4–6)	3 (2–4)	3 (2–6)	0.43
- Closeness – *median (IQR)*	0.29 (0.25–0.31)	0.30 (0.25–0.33)	0.32 (0.29–0.34)	0.66
- Betweenness – *median (IQR)*	158 (129–197)	112 (59–204)	88 (34–100)	0.30

Characteristics of physicians who responded to both pre- and post-survey, categorized by involvement in the design process: those who were involved in the development, those of which ≥ 30% of their network was involved in the development, and those of which < 30% of their network was involved. Two physicians did not finish the social network question and were not included in the social network analyses

*Abbreviations*: *ED* emergency department, *IQR* interquartile range, *n* number

^a^Network measures were calculated based on the question “Who do you discuss problems with at work?”

**Fig. 2:**
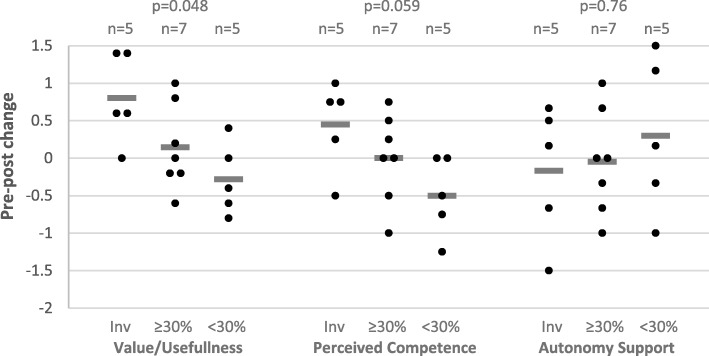
The effects of the performance feedback dashboard on motivation. The effect of the prototype performance feedback dashboard on perceived value/usefulness of making quick disposition decisions, perceived competence in making quick disposition decisions, and perceived autonomy support from emergency department leadership in physicians who were involved in the development (Inv, *n* = 5), those of which ≥ 30% of their network was involved in the development (≥30%, *n* = 7, high exposure), and those with < 30% (< 30%, *n* = 4, low exposure). Horizontal bars represent the group average. Each dot represents a unique observation

When comparing pre- and post-survey responses to the clinical vignette, we did not observe any differences in the percentage of physicians who would order imaging after seeing the prototype dashboard (12/19 pre and 14/19 post; *p* = 0.69). There were also no significant changes in the self-determination theory-based motivation measures; median pre- and post-values for value/usefulness were 4.4 (IQR 4.2–5.2) and 5.0 (IQR 4.0–5.8), respectively (*p* = 0.21); the perceived competence values were 5.5 (IQR 4.3–6.0) and 5.0 (IQR 4.0–6.3), respectively (*p* = 0.88); and the autonomy support values were 5.0 (IQR 2.8–5.7) and 4.0 (IQR 3.0–6.0), respectively (*p* = 0.83). We then assessed changes in sub-groups based on the level of exposure to people involved in the development process as determined by the social network analysis. We found that higher exposure was significantly associated with changes in the perceived value/usefulness of making quick disposition decisions (*p* = 0.048) after seeing the dashboard. The perceived value/usefulness-score of physicians who were directly involved in the development rose by a median of 0.6 points, while the score of physicians with low exposure (< 30% of network was involved) decreased by a median of 0.4 points. Similarly, physicians’ perceived competence in making quick disposition decisions increased in those physicians who were actively involved, and decreased in those with low exposure (NS, *p* = 0.059). No differences between groups were observed in change-scores for autonomy support by ED leadership (Fig. 2). No correlation was observed between the physicians’ perception of the culture in the ED and change scores (Table 3).

**Table 3:** Correlation between climate and outcome measures

	Pre-post (Δ) motivation measures			Dashboard evaluation metrics				
	Δ Value/usefulness	Δ Competence	Δ Autonomy support	Usefulness	Ease of use	Importance of metrics	Ability to affect metrics	Recommend
Teamwork climate	−0.02 (0.95)	0.33 (0.19)	−0.43 (0.07)	−0.07 (0.78)	0.26 (0.30)	0.05 (0.84)	**0.48 (0.046)**	−0.03 (0.92)
Safety climate	0.24 (0.34)	0.41 (0.09)	0.10 (0.69)	**0.55 (0.019)**	**0.50 (0.035)**	**0.54 (0.021)**	**0.71 (0.001)**	**0.52 (0.028)**

Spearman correlation coefficients (*p*-value); Significant values in bold. Motivation measures are derived from Self-Determination Theory

While the importance of the metrics on the dashboard was generally rated highly (median 6 out of 7, IQR 4–6) and physicians reported they could affect the measures (median 6 out of 7, IQR 5–6), the perceived usefulness of the overall dashboard was only rated a median of 4 out of 7 (IQR 3–4.5). The median ease of use rating was 5 out of 7 (IQR 4.3–5.5) and physicians rated the likelihood they would recommend the dashboard to their peers as 6 out of 10 (IQR 5–7). When looking at specific subgroups, we found that physicians with little exposure to those involved in the development process rated the importance of the metrics significantly lower than others (*p* = 0.02, Table 4). A trend towards lower scores for perceived usefulness, ease of use, and likelihood to recommend the dashboard was found in these physicians as well (Table 4). Additionally, moderate correlations between the perceived safety climate and positive evaluations of the dashboard were observed. No correlation was observed between perceived teamwork climate and positive evaluations of the dashboard, except for the ability to affect measures (Table 3).

**Table 4:** Quantitative assessment of the performance feedback dashboard

Outcomes *Median (IQR)*	Overall (*n* = 21)	Involved (*n* = 5)	≥30% of network involved^a^ (*n* = 7)	< 30% of network involved^a^ (*n* = 5)	*p*-value Kruskal-Wallis test
Perceived usefulness (1–7)	4 (3–4.5)	4.3 (4.2–4.5)	4.2 (4–5.7)	3.5 (2–4)	0.36
Perceived ease of use (1–7)	5 (4.3–5.5)	5.5 (5–6.2)	5 (4.3–6)	4.3 (3.8–5)	0.12
Importance of metrics (1–7)					
- LOS	6 (4–6)	6 (5–6)	6 (6–7)	4 (4–4)	0.03
- time to disposition decision	6 (5–6)	6 (6–7)	6 (6–7)	4 (2–4)	0.02
- tests ordered	6 (5–6)	5 (5–6)	6 (6–7)	4 (2–5)	0.03
- metrics overall	6 (4–6)	5 (5–6)	6 (6–7)	3 (2–4)	0.02
Ability to affect metrics (1–7)					
- LOS	5 (4–6)	6 (5–7)	6 (5–6)	5 (4–6)	0.30
- time to disposition decision	6 (5–6)	6 (5–7)	6 (5–6)	6 (5–6)	0.85
- tests ordered	6 (5–7)	6 (5–7)	6 (5–7)	6 (6–6)	0.97
- overall metrics	6 (5–6)	6 (5–7)	6 (5–6)	5 (4–6)	0.30
Recommend (0–10)	6 (5–7)	7 (6–7)	6 (5–8)	3 (2–5)	0.16

Post-survey results of the assessment of the performance feedback dashboard. Separate for physicians who were involved in the development, those of which ≥ 30% of the people they discuss problems with were involved (high exposure), and those with < 30% (low exposure). No network data was available for the 2 physicians who didn’t fill out the pre-survey and for 2 physicians who didn’t fill out the network question

*Abbreviations*: *IQR* interquartile range, *LOS* length of stay, *n* number

^a^Based on the question “Who do you discuss problems with at work?”

Physicians’ comments in the survey were analyzed qualitatively. Several physicians commented that the ability to have measures available would be a “step up”, would “provide an incentive”, or would be “interesting”. The visual design of the performance dashboard was described with words such as “clear”, “easy to understand”, “pleasant to look at”, and “user friendly”, though one physician commented it was “big brother controlling”. Other concerns were related to data accuracy, negative consequences on patient care or work culture, and the effect on the teaching environment. Lastly, while benchmarks were visualized on the dashboard using an interquartile range, several physicians commented that a benchmark would be needed in the dashboard for it to be useful, implying that a more intuitive design to display this information needs to be considered.

## Discussion

This study described the development of a front-end prototype performance feedback dashboard to deliver feedback to attending physicians about their performance with the goal of reducing patient LOS in the ED. We employed a human-centered design approach to ensure we developed a dashboard that fits the end-users needs. This approach guided the selection of measures, development of functionality, and visualization of data. During the eleven interviews performed as part of the human-centered design process, a variety of barriers were identified that we attempted to address in the design. Additionally, we found preliminary evidence that the social network in which the intervention is developed and implemented is likely to be of key importance to the adoption of the intervention and the achievement of the intended changes in physicians’ practice patterns.

The involvement of end-users in the design of the dashboard allowed us to select relevant measures. Particularly, it helped us select measures that were perceived as actionable and under control of the physician. It also helped identify appropriate comparisons and clear visualizations. The multiple interviews before and during development provided insights about the importance of having drilldown functionality, both to increase trust in the data as well as to correct for confounding variables such as patient acuity. While we were able to address a variety of concerns that came up during the design process, other issues were not sufficiently addressed according to post-survey results. Interviewees expressed concerns about the application of a performance-feedback tool in an educational setting, in which they also have the responsibility to teach residents. This is consistent with prior findings that clear goals need to be aligned with audit and feedback interventions [14]. Another recurring barrier was the lack of trust in EMR data. This too is consistent with prior studies, and a variety of strategies are available to improve the accuracy of, and trust in, EMR data for quality improvement purposes [42, 43].

We showed preliminary evidence that the ED/workplace culture may be important for the adoption of technology. Physicians who reported higher scores on questions about safety climate based on questions such as “I would feel safe being treated here as a patient” and “The culture in this clinical area makes it easy to learn from the errors of others,” also reported higher scores for acceptability of the dashboard. This suggests that nurturing a culture that supports learning from errors is important for acceptance of performance feedback. Additionally, we showed data that suggests that the involvement of end-users in the design can lead to higher acceptability and increased motivation to change. Not only were those involved in the design more likely to report higher acceptance scores, this effect also appeared to be disseminated through their social network; conversely, physicians with little exposure to those involved in the design of the dashboard reported lower acceptance of the dashboard compared to those who had higher levels of exposure. Similarly, the dashboard had a positive effect on motivation of those involved in the design and a negative effect on those with little exposure. These findings are consistent with other studies showing that social networks have important roles in dissemination of innovation and behavior change [22, 27–29]. This implies that it might be important to strategically select the people involved in the design process based on their position in the social network.

While these preliminary findings offer useful insights, the sample size is small and with only 19 of 56 participants responding to both surveys, there is likely a selection bias. Therefore, these results should not be interpreted as a proof of concept, but rather as preliminary evidence that needs to be further explored in future studies. Additionally, physicians who were included in the design process had more experience than those who were not involved and had a more central position in the ED social network. This is likely due to our purposive sample selection strategy along with the selection bias introduced by the participation of only about half of the physicians that were invited. We purposefully excluded physicians with less than 2 years of experience in this ED because we expected those to have less insight regarding the needs and workflows of the ED. Moreover, our study only included attending physicians at a single site, limiting generalizability to other settings and other healthcare providers such as nurse practitioners. Furthermore, while we were able to test the effect of this prototype dashboard on physicians’ motivation to make faster disposition decisions, the prototype did not include real, personalized data which likely decreased the observed impact.

Lastly, we would like to acknowledge that caution should be exercised when attempting to implement a performance dashboard such as the one developed in this study. Both intended and unintended consequences should be monitored closely, for example through the inclusion of balancing measures. This is especially important when appropriateness measures are not readily available for metrics such as test utilization and LOS in the ED; reductions in test ordering may not always be appropriate and reductions in LOS might not always improve quality of care even if they may increase patient satisfaction. While we included 72-h return rates as a balancing measure on the dashboard, several other potential unintended consequences were identified in the interview process, including physician stress levels, dehumanization of the patient experience, effects on teaching, and effects on quality and safety of care.

## Conclusions

In summary, this small pilot study in a single ED showed the feasibility of designing a performance feedback dashboard using a human-centered design approach over the course of 8 months. Additionally, we showed preliminary evidence suggesting that both the culture and social network can be leveraged to facilitate the adoption and enhance the effect of such an intervention. A social network analysis prior to the initiation of the human-centered design process could help identify the right stakeholders to involve in this process and increase the likelihood of successful implementation. This evidence, along with the observation that data quality and unclear goals are barriers to acceptance, shows that implementation of an audit and feedback intervention cannot happen in a vacuum; this type of intervention requires close collaboration of senior leadership, designers, researchers, IT, and end-users.

## Additional files

Additional file 1: Interview Guides. The interview guides used in the human-centered design process. Three interview guides are included; the interview guide used for leadership interviews, the interview guide for attending physician interviews for the first round of interviews, and the interview guide used during the iterative improvement process. (DOCX 32 kb)

Additional file 2: Pre- and Post Surveys. The complete pre- and post-surveys used in this study. The surveys were administered online. (DOCX 62 kb)

Additional file 3: Supplemental Tables and Figures. The supplemental tables and figures references throughout the paper. (DOCX 272 kb)

## Abbreviations

ED: Emergency Department
EMR: Electronic medical record
LAC+USC: Los Angeles County + University of Southern California
LOS: Length of stay
USC: University of Southern California
