# Supplementary material for: Involving end-users in the design of an audit and feedback intervention in the emergency department setting – a mixed methods study
Source: BMC Health Serv Res. 2019 Apr 29;19:270. doi: 10.1186/s12913-019-4084-3 (PMC6489283; doi:10.1186/s12913-019-4084-3)
Supplement: Supplementary file 1 — Interview Guides. The interview guides used in the human-centered design process. Three interview guides are included; the interview guide used for leadership interviews, the interview guide for attending physician interviews for the first round of interviews, and the interview guide used during the iterative improvement process. (DOCX 32 kb) [file 12913_2019_4084_MOESM1_ESM.docx]

**INTERVIEW GUIDES**

**Emergency Department Leadership Interview Guide -** Page 1
 **Attending Physician Interview Guide -** Page 4 **Iterative Improvement Interview Guide -** Page 7

**Emergency Department Leadership Interview Guide**

# Objectives

1. Identify Emergency Department leadership’s goals for a performance improvement dashboard
2. Identify the intended end-users of the dashboard
3. Identify the metrics to be used to evaluate the success of the dashboard

# Discussion Guide

## Section 1. Introduction/Background (3 min)

Hi Dr. ____, thank you for your time today. We’re this interview should take less than 45 minutes

As you may already know, we are working on a project to design a performance improvement dashboard for the emergency department at LAC+USC. Our goal is to design a dashboard that helps facilitate improvement and to also measure the behavioral effects of the dashboard on its end-users. As such, we are speaking with you in order to understand the ED leadership’s goals with such a dashboard, its intended end users, and the metrics to be used to evaluate its success.

Before we begin, however, we’d like to start off with some general background questions about yourself.

1. How long have you been practicing as a physician?
2. What is your current function or role in the Emergency Department?
3. How long have you been in this role?
4. What are your primary responsibilities in your current role?

## Section 2. Goals with Performance Improvement Dashboard (40min)

As we mentioned earlier, we plan to create a performance improvement dashboard that will further facilitate improvement in the emergency department at LAC+USC. Before we go into more details about the dashboard, we’d like to learn more about current operational goals in the ED.

1. Does the emergency department currently have strategic goals related to ED functions or operations for the current or future calendar years?
   1. What are these goals?
2. Are any of these goals are tied to performance metrics currently measured in the ED?
   1. Which ones?
   2. *[If not readily apparent]* How are they tied together?
3. Are there performance goals that will require new metrics and tools, if so which ones?
4. Do you currently have a performance dashboard? If so how does it relate to your current and future goals?
   1. Which of these goals would you consider most important?
   2. Why do you consider these goals to be the most important?
5. Who do you intend to be the users of this dashboard?
   1. Who will be providing data? What data will they provide?
   2. Who will be using results? Which results will they use?
   3. *[If not mentioned]* What about residents vs. attending physicians?
   4. Within residents, what about junior vs. senior residents?
   5. What roles will they play?
6. Who do you intend to be the primary users of the information the dashboard would provide?
   1. How would you like them to use thes results?
   2. How would the use of the dashboard fit into their daily work? *[ask them to elaborate until you understand how often they would use it]*
   3. What information do you want shared with these users through the dashboard?
   4. How would you envision behavior changing as a result of the use of the dashboard?
   5. Is there any information that you want to track but do not want to share with these users?
      1. If so, what information? Why?
7. Are there any other intended users for this dashboard?
   1. If different from the users we discussed earlier, how would you like them to use dashboard?
   2. What information do you want shared with these users through the dashboard?
   3. What behaviors do you want to influence with the dashboard?
   4. What outcomes are you looking for with this dashboard with these users?
   5. Is there any information that you want to track but do not want to share with these users?
      1. If so, what information? Why?
8. Do you want each of the user groups to use the dashboard similarly or differently?
   1. *[If similarly]* Why similarly?
   2. *[If differently]* Why differently?
   3. *Probe on differences between attendings vs. senior residents vs. junior residents/interns if applicable.*
9. How would you measure or evaluate the success of the dashboard?
   Probe on qualitative vs. quantitative measures
   1. What qualitative measures would you use?
   2. What quantitative measures would you use?
10. How do you think hospital leadership will view the dashboard?
11. Are there any other key stakeholders besides direct users that would be directly impacted by the dashboard?
    1. Any other stakeholders who would be interested in the dashboard?

## Section 3. Performance Metrics (15 min)

We’ve spoken at length about the goals of a performance improvement dashboard for the ED. Let’s talk about the metrics to be included in the dashboard.

1. What are the key performance metrics that are tracked at a departmental level?
   *Probe on the following if not initially mentioned.*
   1. Length of stay?
   2. Time to evaluation by physician?
   3. Time to disposition decision?
   4. Time to treatment?
   5. Time to consultation?
   6. Lab or radiology turnaround time?
   7. Number of patients seen?
   8. Number of patients waiting?
   9. Other metrics?
2. Which of these are most important for the ED?
   1. What makes them important? How will you use them?
3. What are the key performance metrics that are tracked at a physician level?
   1. Which of these are relevant at a departmental level?
   2. Are any of these metrics not tracked at a departmental level?
      1. If so, why?
   3. Which of these would you rate are most important for evaluating physicians’ performance?
   4. Which of these would you rate most easily influenced by physicians? Why?
4. Are there any metrics that are not currently being tracked that you believe are important to evaluate a provider’s performance?
   1. Why are these metrics not currently being tracked?
   2. Are there any surrogate markers that can be used in lieu of these metrics?

## Section 4. Wrap-up (2 min)

Thank you for your time today.

1. Is there anything else you would like to share with us before we conclude the interview?
2. Do you have any questions for us?

Thank you for your time.

**Attending Physician Interview Guide**

# Objectives

1. Assess end user overall perception of a performance improvement dashboard
2. Identify and understand perceived barriers to change, and impact on intrinsic motivation
3. Collect and assess feedback for desired functions, features, and metrics for dashboard
4. Assess the perceived relevance, actionability, and risk of bias of potential performance metrics

# Discussion Guide

## Section 1. Introduction/Background (3 min)

Hi Dr. ____, thank you for your time today. We’re hoping this interview will last about an hour.

We are working with the Dr. XXXXXX and other Emergency Department leadership to design a performance improvement dashboard for the ED at LAC+USC. Our goal is to design a dashboard that helps facilitate understanding and improvement and to also measure the effects of the dashboard on physicians such as yourself. As such, we plan to spend the next hour to learn more about your thoughts and perceptions about such a dashboard and also to solicit suggestions or feedback for the future dashboard.

Before we begin, we’d like to start off with some general background questions about yourself.

1. How long have you been practicing as a physician?
2. What is your current function or role in the Emergency Department?
3. How long have you been in this role?
4. What are your primary responsibilities in your current role?
5. Are you currently using any “tools” that help you monitor your performance? If so, what are they?

## Section 2. Perceptions of Performance Improvement Dashboard (10 min)

As we mentioned earlier, we plan to create a performance improvement dashboard that will further facilitate improvement in the emergency department at LAC+USC. The dashboard is intended to improve [INSERT GOALS DERIVED FROM LEADERSHIP INTERVIEW] by showing you data that could help you improve your performance towards this goal.

1. Do you have any previous experience with a performance dashboard? If so
   1. Can you describe the dashboard?
   2. What were the positive aspects of the dashboard, if any?
   3. What were the negative aspects of the dashboard, if any?
   4. How would you have improved it?
2. What are your initial thoughts about the prospects of such a performance dashboard?
   1. Do you see this as an overall welcome or unwelcomed tool?
      1. Why?
   2. What would make this an unwelcome tool?
      1. What would make this a welcome tool?
3. What key concerns do you have with having such a dashboard?

*Probes: Effect on teaching responsibilities? Quality of data? Actionability? Fairness?*

- 1. What would alleviate such concerns?
     *[Use their words and ask them to be specific. If they continue to describe abstract problems or benefits ask them to provide a hypothetical example.]*

*[Use Q4 and Q5 to probe on specific part of workflow where this dashboard could be utilized]*

1. Would you use such a dashboard?
   1. Why or why not?
2. How would you use the dashboard?
   1. Are there specific activities that you would use this dashboard in conjunction with?
   2. How would you use this to support these activities?
3. What are the key functions and features you would like to see in the dashboard?
   1. How important are each of these functions and features to you?
      1. What makes them important?
4. What are some key challenges you anticipate in using such a dashboard?
5. A dashboard could you public or private - which one do you think is most appropriate?
   1. Which information would you make public?
   2. Which information would be private?

## Section 3. Performance Metrics (30 min)

Let’s now spend some time talking about potential metrics and data points to be included in the dashboard.

*Use the table below as a working space to document the responses below.*

| **Metric description** | **Potential benefit** |  | **Additional Comments** |
| --- | --- | --- | --- |
|  |  |  |  |
|  |  |  |  |
|  |  |  |  |
|  |  |  |  |
|  |  |  |  |
|  |  |  |  |

1. What are some metrics you would envision to be included in a performance improvement dashboard? *(fill out in table above)*
2. What are some metrics that you would like to track for yourself? *(fill out in table above)*
3. Are you currently measuring and monitoring in any capacity any performance measures for yourself?
   1. Why or why not?
4. What are some measures that you think would help you improve as a physician if you were able to monitor and understand them? *(fill out in table above)*
5. How would you use these metrics to improve your performance?
6. At what level of granularity would the performance need to be in order to be helpful?
   1. Per patient?
   2. Daily/weekly/monthly summaries?
   3. Are there any metrics that should be tracked more frequently?
7. Below is a list of potential metrics that are currently tracked at a departmental level.

*Use the table below as a working space to document the responses above.*

| **Metric** | **Actionability** | **Relevance** | **Frequency** |
| --- | --- | --- | --- |
| Length of stay |  |  |  |
| Time to evaluation by physician |  |  |  |
| Time to disposition decision |  |  |  |
| Number of patients seen |  |  |  |
| Number of patients waiting |  |  |  |
| % of patients admitted |  |  |  |
| % LWBS/LBTC/AMA |  |  |  |

1. Which of these metrics do you feel you could influence the most? And which one the least? *(Use the table above to document the responses.)*
2. Which of these metrics do you feel is the most relevant to your performance as physician? And which one the least? *(Use the table above to document the responses.)*
3. How frequently should [MOST ACTIONABLE METRIC FROM Q9] be tracked? And [MOST RELEVANT METRIC FROM Q10]?
4. Are there any measures that should be tracked more/less frequent? Why?
5. Imagine you could drastically improve any one of these metrics overnight, which metric would you choose? Why?

## Section 4. Wrap-up (2min)

Thank you for your time today.

1. Is there anything else you would like to share with us before we conclude the interview?
2. Do you have any questions for us?

**Iterative Improvement Interview Guide**

# Objectives

1. Assess end user overall perception of the designed performance improvement dashboard
2. Identify and understand perceived barriers to change and impact on intrinsic motivation
3. Collect and assess feedback for desired functions and features
4. Assess the perceived relevance, actionability, and risk of bias of included performance metrics

# Discussion Guide

## Section 1. Introduction/Background (3 min)

Hi Dr. ____, thank you for your time today. We’re hoping this interview will last less than 45 minutes. Let me know if you’d like to stop or take a break at any point.

We are working with the Dr. XXXX and other Emergency Department leadership to design a performance improvement dashboard for the ED at LAC+USC. Our goal is to design a dashboard that helps facilitate understanding and improvement and to also measure the effects of the dashboard on physicians such as yourself. We developed a prototype dashboard that we wish to get your feedback on. As such, we plan to spend the next 45 minutes to learn more about your thoughts and perceptions about this dashboard. We welcome both positive and negative feedback. If you do have concerns it’s important that you share them and any suggestions you may have.

## Section 2. General perceptions of the Performance Improvement Dashboard (20 min)

As we mentioned earlier, we aim to create a performance improvement dashboard that will further facilitate improvement in the emergency department at LAC+USC. The dashboard is intended to improve [INSERT GOALS DERIVED FROM LEADERSHIP INTERVIEW] by showing you data that could help you improve your performance towards this goal. *[Show the prototype]* Based on the prior interviews this is the dashboard we developed

1. Please take a look at this performance improvement dashboard. What are your initial impressions?
   1. [if not immediately clear] How do you feel about this tool?
      1. Is there anything in particular about the tool that makes you think this way? [Probe on specific design features or elements]
   2. What do you think is the purpose of this dashboard?
      1. What makes you think this way? [Probe on specific design features or elements]
   3. Who do you think this this tool was made for?
      1. What about the tool gives you this impression?
      2. [If not ED physician] What about for ED physicians? Why or why not?
   4. Does this dashboard look easy to use? Why or why not?
      1. Are there any specific features or functions that make you feel this way?
2. Would you use this dashboard?
   1. Why or why not?
   2. Are there any specific functions in this dashboard that you would use?
      1. Which functions? [Ask follow-up questions to understand the context of use and the goal the physician would have]
      2. How would you use them?
      3. What would you use them for? [Probe on the context of use and the goal the physician would have]
   3. Is there anything in this tool that you would not use?
      1. Any specific functions or features?
      2. Why would you not use these functions?
   4. Is there anything about this tool that concerns you?

[Keep the respondent grounded on **their** own impressions and uses of the tool, rather than allowing them to make generalized statements about “physicians’” impressions and uses of the tool]

1. How do you envision the introduction of this tool to the ED?
   1. Would this be an overall welcome or unwelcomed tool?
      1. What makes this a welcome tool?
      2. What makes this an unwelcomed tool?
      3. What challenges do you foresee in introducing this tool to the ED?
      4. What are some ways you would mitigate these challenges?
2. What are your greatest concerns about having such a tool in the ED?
   1. Why are they a concern to you?
      1. [Probe the following if not addressed]:
         1. Effect on teaching responsibilities?
         2. Quality of data?
            1. Risk of bias?
         3. Actionability?
         4. Fairness?
   2. What would alleviate such concerns?
      *[Use their words and ask them to be specific. If they continue to describe abstract problems or benefits ask them to provide a hypothetical example.]*
3. What do you see as your greatest barrier to using this tool? Why?
   1. What would lower this barrier?
4. We’ve talked a lot about some of the challenges and barriers to this tool, but do you see any positive aspects to this dashboard?
   1. What makes these aspects positive?
   2. What do you personally like about this dashboard? Why?

## Section 3. Performance Metrics (20 min)

I’d like to switch gears now and spend some time talking about the data and metrics included in the performance summary tool.

*Use the table below as a working space to document the responses above.*

| **Metric** | **Actionability** | **Relevance** | **Frequency** | **Concerns** |
| --- | --- | --- | --- | --- |
|  |  |  |  |  |
|  |  |  |  |  |
|  |  |  |  |  |
|  |  |  |  |  |
|  |  |  |  |  |
|  |  |  |  |  |
|  |  |  |  |  |

1. Which of these metrics do you feel is the most relevant to your performance as physician? *(Use the table above to document the responses.)*
   1. Are there any metrics that are more relevant to you?
2. Which of these metrics do you feel is the least relevant to your performance as physician? *(Use the table above to document the responses.)*
3. Which of these metrics do you feel you could influence the most? Why?
   *(Use the table above to document the responses.*
4. Which of these metrics do you feel you could influence the least? Why?
   *(Use the table above to document the responses.)*
5. The dashboard shows *[daily/weekly/monthly]* data. Do you think that is the right frequency for *[MOST ACTIONABLE METRIC FROM Q1]*? Why?
6. What is the right frequency to display data for *[MOST RELEVANT METRIC FROM Q2]?*
7. Are there any measures that should be tracked more or less frequently? Why?
8. Imagine you could snap your fingers and improve any one of these metrics overnight, which metric would you choose? Why?
   *(Use the table above to document the responses).*
   1. *[Probe if respondent selects an unusual or unexpected metric such as:*
      1. *Metrics which respondent said they have little influence over*
      2. *Metrics with low relevance +/- low influence*

## Section 4. Wrap-up (2 min)

Thank you for your time today.

1. Is there anything else you would like to share with us before we conclude the interview?
2. Do you have any questions for us?
