# Supplementary material for: Involving end-users in the design of an audit and feedback intervention in the emergency department setting – a mixed methods study
Source: BMC Health Serv Res. 2019 Apr 29;19:270. doi: 10.1186/s12913-019-4084-3 (PMC6489283; doi:10.1186/s12913-019-4084-3)
Supplement: Supplementary file 2 — Pre- and Post Surveys. The complete pre- and post-surveys used in this study. The surveys were administered online. (DOCX 62 kb) [file 12913_2019_4084_MOESM2_ESM.docx]

**PRE- AND POST SURVEYS**

**Pre-survey -** page 1

**Post-survey** - page 7

Pre-survey

**Last updated 11/22/2017**

# **Section 1. Demographics**

**1Q1.** Approximately how many years have you been practicing medicine post-residency? (whole numbers only)

*Allow integer values (0-99) only with no decimal places.*

| **[*FREE TEXT BOX]*** |
| --- |

**1Q2.** Approximately how many years have you been practicing medicine at LAC+USC?

*Allow integer values (0-99) only with no decimal places.*

| **[*FREE TEXT BOX]*** |
| --- |

# **Section 2. Clinical Vignette**

A 14-year-old boy presents via ambulance 1 h following a collision

with another player where they collided heads during a rugby

game. He was knocked out for 2 min. There was no reported

seizure activity. Currently he is complaining of a headache and

nausea. Medical history reveals asthma and a concussion 2 years

prior, medications = ventolin p.r.n. On examination he has a GCS

of 13 (Eye 3, Movement 6, Verbal 4), no signs of basal skull

fracture, hasa2cmtender haematoma on his forehead, pupils are

equal and reactive to light, he cannot recall the incident or the

game around it, and is not oriented to time or place.

A 14-year-old boy presents via ambulance 1 h following a collision

with another player where they collided heads during a rugby

game. He was knocked out for 2 min. There was no reported

seizure activity. Currently he is complaining of a headache and

nausea. Medical history reveals asthma and a concussion 2 years

prior, medications = ventolin p.r.n. On examination he has a GCS

of 13 (Eye 3, Movement 6, Verbal 4), no signs of basal skull

fracture, hasa2cmtender haematoma on his forehead, pupils are

equal and reactive to light, he cannot recall the incident or the

game around it, and is not oriented to time or place.

A 14-year-old boy presents via ambulance 1 h following a collision

with another player where they collided heads during a rugby

game. He was knocked out for 2 min. There was no reported

seizure activity. Currently he is complaining of a headache and

nausea. Medical history reveals asthma and a concussion 2 years

prior, medications = ventolin p.r.n. On examination he has a GCS

of 13 (Eye 3, Movement 6, Verbal 4), no signs of basal skull

fracture, hasa2cmtender haematoma on his forehead, pupils are

equal and reactive to light, he cannot recall the incident or the

game around it, and is not oriented to time or place.

A 14-year-old boy presents via ambulance 1 h following a collision

with another player where they collided heads during a rugby

game. He was knocked out for 2 min. There was no reported

seizure activity. Currently he is complaining of a headache and

nausea. Medical history reveals asthma and a concussion 2 years

prior, medications = ventolin p.r.n. On examination he has a GCS

of 13 (Eye 3, Movement 6, Verbal 4), no signs of basal skull

fracture, hasa2cmtender haematoma on his forehead, pupils are

equal and reactive to light, he cannot recall the incident or the

game around it, and is not oriented to time or place.

A 14-year-old boy presents via ambulance 1 h following a collision

with another player where they collided heads during a rugby

game. He was knocked out for 2 min. There was no reported

seizure activity. Currently he is complaining of a headache and

nausea. Medical history reveals asthma and a concussion 2 years

prior, medications = ventolin p.r.n. On examination he has a GCS

of 13 (Eye 3, Movement 6, Verbal 4), no signs of basal skull

fracture, hasa2cmtender haematoma on his forehead, pupils are

equal and reactive to light, he cannot recall the incident or the

game around it, and is not oriented to time or place.

Below is a clinical vignette for a patient that presents to your emergency department. Please read the vignette and answer the following questions.

A 14-year-old boy presents via ambulance 1 h following a collision with another player where they collided heads during a rugby game. He was knocked out for 2 min. There was no reported seizure activity. Currently he is complaining of a headache and nausea. Medical history reveals asthma and a concussion 2 years prior, medications = ventolin p.r.n. On examination he is moving all extremities purposefully, is speaking in clear sentences, but doesn’t remember the event. He has no signs of basal skull fracture, has a 2cm tender hematoma on his forehead, pupils are equal and reactive to light, he cannot recall the incident or the game around it, and is not oriented to time or place.

**2Q1.** Would you order diagnostic imaging immediately for this patient?

| 1 | O | Yes |
| --- | --- | --- |
| 0 | O | No |

**[SHOW 2Q2 IF 2Q1 = “Yes”]**

**2Q2.** What diagnostic imaging would you order for your immediate plan? (select any that apply)

|  |  | **Diagnostic Imaging Choice (select any that apply)**  ***[RANDOMIZE ITEMS BELOW]*** |  |
| --- | --- | --- | --- |
| 1 | □ | Skull X-ray |  |
| 2 | □ | CT scan |  |
| 3 | □ | MRI scan |  |
| 4 | □ | Other (please specify: ______) | ***[FREE TEXT BOX]*** |

**[SHOW 2Q3 IF 2Q1 = “No”]**

**2Q3.** What is your immediate plan for this patient? (select any that apply)

|  |  | **Management Choice (select any that apply)**  ***[RANDOMIZE ITEMS BELOW]*** |  |
| --- | --- | --- | --- |
| 1 | □ | Discharge home now |  |
| 2 | □ | Give analgesia |  |
| 4 | □ | Observation in ED / SOU / hospital for ___ hours (please specify # of hours): | ***[FREE TEXT BOX]*** |
| 5 | □ | Admit to hospital |  |
| 6 | □ | Refer for follow-up with outpatient provider |  |
| 7 | □ | Other (please specify: ______) | ***[FREE TEXT BOX]*** |

**[SHOW 2Q5 IF 2Q1 = “Yes”]**

**2Q5.** The results of the diagnostic imaging you order are unremarkable. What is your management plan now? (select any that apply)

|  |  | **Management Choice (select any that apply)**  **[RANDOMIZE OPTIONS]** |  |
| --- | --- | --- | --- |
| 1 | □ | Discharge home now |  |
| 2 | □ | Give analgesia |  |
| 3 | □ | Order more diagnostic imaging |  |
| 4 | □ | Observation in ED / SOU / hospital for ___ hours (please specify # of hours): | ***[FREE TEXT BOX]*** |
| 5 | □ | Admit to hospital |  |
| 6 | □ | Refer for follow-up with outpatient provider |  |
| 7 | □ | Other (please specify: ______) | ***[FREE TEXT BOX]*** |

# **Section 3. Motivation to improve**

For each of the following statements, please indicate how true it is for you using the following scale:

| **1** | **2** | **3** | **4** | **5** | **6** | **7** |
| --- | --- | --- | --- | --- | --- | --- |
| Not at all true |  |  | Somewhat true |  |  | Very true |

**3Q1. Value/Usefulness and Perceived Competence**

|  |  | **1** | **2** | **3** | **4** | **5** | **6** | **7** |
| --- | --- | --- | --- | --- | --- | --- | --- | --- |
|  | **Statement *[RANDOMIZE ITEMS BELOW]*** | Not at all true |  |  | Somewhat true |  |  | Very true |
| 1 | I think that making quick disposition decisions is useful to improve patient outcomes. | O | O | O | O | O | O | O |
| 2 | I think it is important to make quick disposition decisions for patient satisfaction. | O | O | O | O | O | O | O |
| 3 | I think making quick disposition decisions could help me deliver better care. | O | O | O | O | O | O | O |
| 4 | I believe making quick disposition decisions could be beneficial to me. | O | O | O | O | O | O | O |
| 5 | I think making quick disposition decisions is important. | O | O | O | O | O | O | O |
| 6 | I think I am pretty good at making quick disposition decisions. | O | O | O | O | O | O | O |
| 7 | I think I do pretty well at making quick disposition decisions, compared to others. | O | O | O | O | O | O | O |
| 8 | I am satisfied with how quickly I make disposition decisions. | O | O | O | O | O | O | O |
| 9 | I feel pretty skilled in making quick disposition decisions | O | O | O | O | O | O | O |

**3Q3.** **Autonomy Support**

|  |  | **1** | **2** | **3** | **4** | **5** | **6** | **7** |
| --- | --- | --- | --- | --- | --- | --- | --- | --- |
|  | **Statement *[RANDOMIZE ITEMS BELOW]*** | Not at all true |  |  | Somewhat true |  |  | Very true |
| 1 | I feel that the ED medical directors provide me choices and options. | O | O | O | O | O | O | O |
| 2 | I feel understood by the ED medical directors. | O | O | O | O | O | O | O |
| 4 | The ED medical directors convey confidence in my ability to do well at my job. | O | O | O | O | O | O | O |
| 7 | The ED medical directors encourage me to ask questions. | O | O | O | O | O | O | O |
| 10 | The ED medical directors listen to how I would like to do things. | O | O | O | O | O | O | O |
| 14 | The ED medical directors try to understand how I see things before suggesting a new way to do things. | O | O | O | O | O | O | O |

# **Section 4. Work environment**

Rate your level of agreement with the following statements with respect to the emergency department using the scale below.

| **1** | **2** | **3** | **4** | **5** | **N/A** |
| --- | --- | --- | --- | --- | --- |
| Disagree strongly | Disagree slightly | Neutral | Agree slightly | Agree strongly | Not applicable |

**4Q1. Teamwork Climate**

Indicate your level of agreement with each of the statements:

|  |  | **1** | **2** | **3** | **4** | **5** | **N/A** |
| --- | --- | --- | --- | --- | --- | --- | --- |
|  | **Statement** | Disagree strongly | Disagree slightly | Neutral | Agree Slightly | Agree Strongly | Not Applicable |
| 1 | Nurse input is well received in this clinical area. | O | O | O | O | O | O |
| 2 | In this clinical area, it is difficult to speak up if I perceive a problem with patient care. | O | O | O | O | O | O |
| 3 | Disagreements in this clinical area are resolved appropriately (i.e., not who is right, but what is best for the patient). | O | O | O | O | O | O |
| 4 | I have the support I need from other personnel to care for patients. | O | O | O | O | O | O |
| 5 | It is easy for personnel here to ask questions when there is something that they do not understand. | O | O | O | O | O | O |
| 6 | The physicians and nurses here work together as a well-coordinated team. | O | O | O | O | O | O |

**4Q2. Safety Climate**

Indicate your level of agreement with each of the statements:

|  |  | **1** | **2** | **3** | **4** | **5** | **N/A** |
| --- | --- | --- | --- | --- | --- | --- | --- |
|  | **Statement** | Disagree strongly | Disagree slightly | Neutral | Agree Slightly | Agree Strongly | Not Applicable |
| 1 | I would feel safe being treated here as a patient. | O | O | O | O | O | O |
| 2 | Medical errors are handled appropriately in this clinical area. | O | O | O | O | O | O |
| 3 | I know the proper channels to direct questions regarding patient safety in this clinical area. | O | O | O | O | O | O |
| 4 | I receive appropriate feedback about my performance. | O | O | O | O | O | O |
| 5 | In this clinical area, it is difficult to discuss errors. | O | O | O | O | O | O |
| 6 | I am encouraged by my colleagues to report any patient safety concerns I may have. | O | O | O | O | O | O |
| 7 | The culture in this clinical area makes it easy to learn from the errors of others. | O | O | O | O | O | O |

# **Section 5. Social Network**

**Now we’d like to ask you about your professional network at work. Please answer the following questions to the best of your ability. As a reminder, your responses will be kept anonymous and will only be reported in aggregate.**

**5Q1.** Who do you go to for advice about difficult clinical decisions? (select up to 7)

***[MULTISELECT OPTION]***

| Name 1 | Name 22 | Name 43 |
| --- | --- | --- |
| Name 2 | Name 23 | Name 44 |
| Name 3 | Name 24 | Name 45 |
| Name 4 | Name 25 | Name 46 |
| Name 5 | Name 26 | Name 47 |
| Name 6 | Name 27 | Name 48 |
| Name 7 | Name 28 | Name 49 |
| Name 8 | Name 29 | Name 50 |
| Name 9 | Name 30 | Name 51 |
| Name 10 | Name 31 | Name 52 |
| Name 11 | Name 32 | Name 53 |
| Name 12 | Name 33 | Name 54 |
| Name 13 | Name 34 | Name 55 |
| Name 14 | Name 35 | Name 56 |
| Name 15 | Name 36 | Name 57 |
| Name 16 | Name 37 | Name 58 |
| Name 17 | Name 38 | Name 59 |
| Name 18 | Name 39 | Name 60 |
| Name 19 | Name 40 | Name 61 |
| Name 20 | Name 41 | Name 62 |
| Name 21 | Name 42 | Name 63 |
| Other (Please specify: ______) | | |

**5Q2.** Who do you discuss problems with at work? (select up to 7)

***[MULTISELECT OPTION]***

| Name 1 | Name 22 | Name 43 |
| --- | --- | --- |
| Name 2 | Name 23 | Name 44 |
| Name 3 | Name 24 | Name 45 |
| Name 4 | Name 25 | Name 46 |
| Name 5 | Name 26 | Name 47 |
| Name 6 | Name 27 | Name 48 |
| Name 7 | Name 28 | Name 49 |
| Name 8 | Name 29 | Name 50 |
| Name 9 | Name 30 | Name 51 |
| Name 10 | Name 31 | Name 52 |
| Name 11 | Name 32 | Name 53 |
| Name 12 | Name 33 | Name 54 |
| Name 13 | Name 34 | Name 55 |
| Name 14 | Name 35 | Name 56 |
| Name 15 | Name 36 | Name 57 |
| Name 16 | Name 37 | Name 58 |
| Name 17 | Name 38 | Name 59 |
| Name 18 | Name 39 | Name 60 |
| Name 19 | Name 40 | Name 61 |
| Name 20 | Name 41 | Name 62 |
| Name 21 | Name 42 | Name 63 |
| Other (Please specify: ______) | | |

Post-survey

**Last updated 12/18/2017**

# **Section 1. Demographics**

**1Q1.** Approximately how many years have you been practicing medicine post-residency?

*Allow integer values (0-99) only with no decimal places.*

| **[*FREE TEXT BOX]*** |
| --- |

**1Q2.** Approximately how many years have you been practicing medicine at LAC+USC?

*Allow integer values (0-99) only with no decimal places.*

| **[*FREE TEXT BOX]*** |
| --- |

**1Q3.** Below is an image of the prototype of a tool developed to provide performance feedback for emergency medicine physicians. The data presented is a mock-up and is meant to be an example of what could be shown to physicians. Please review the prototype. You can click on the data for more information. We will ask you more questions later in this survey.

**[SHOW SCREENSHOT OF EMAIL MOCKUP]**

**1Q4.** Below is an example of a more detailed summary of individual metrics which can be customized to your individual needs. Again, the data presented is a mock-up and is meant to be an example of what could be shown to physicians.

**[SHOW SCREENSHOT OF INTERACTIVE MOCKUP]**

# **Section 2. Clinical Vignette**

1. A 14-year-old boy presents via ambulance 1 h following a collision
2. with another player where they collided heads during a rugby
3. game. He was knocked out for 2 min. There was no reported
4. seizure activity. Currently he is complaining of a headache and
5. nausea. Medical history reveals asthma and a concussion 2 years
6. prior, medications = ventolin p.r.n. On examination he has a GCS
7. of 13 (Eye 3, Movement 6, Verbal 4), no signs of basal skull
8. fracture, hasa2cmtender haematoma on his forehead, pupils are
9. equal and reactive to light, he cannot recall the incident or the
10. game around it, and is not oriented to time or place.
11. A 14-year-old boy presents via ambulance 1 h following a collision
12. with another player where they collided heads during a rugby
13. game. He was knocked out for 2 min. There was no reported
14. seizure activity. Currently he is complaining of a headache and
15. nausea. Medical history reveals asthma and a concussion 2 years
16. prior, medications = ventolin p.r.n. On examination he has a GCS
17. of 13 (Eye 3, Movement 6, Verbal 4), no signs of basal skull
18. fracture, hasa2cmtender haematoma on his forehead, pupils are
19. equal and reactive to light, he cannot recall the incident or the
20. game around it, and is not oriented to time or place.
21. A 14-year-old boy presents via ambulance 1 h following a collision
22. with another player where they collided heads during a rugby
23. game. He was knocked out for 2 min. There was no reported
24. seizure activity. Currently he is complaining of a headache and
25. nausea. Medical history reveals asthma and a concussion 2 years
26. prior, medications = ventolin p.r.n. On examination he has a GCS
27. of 13 (Eye 3, Movement 6, Verbal 4), no signs of basal skull
28. fracture, hasa2cmtender haematoma on his forehead, pupils are
29. equal and reactive to light, he cannot recall the incident or the
30. game around it, and is not oriented to time or place.
31. A 14-year-old boy presents via ambulance 1 h following a collision
32. with another player where they collided heads during a rugby
33. game. He was knocked out for 2 min. There was no reported
34. seizure activity. Currently he is complaining of a headache and
35. nausea. Medical history reveals asthma and a concussion 2 years
36. prior, medications = ventolin p.r.n. On examination he has a GCS
37. of 13 (Eye 3, Movement 6, Verbal 4), no signs of basal skull
38. fracture, hasa2cmtender haematoma on his forehead, pupils are
39. equal and reactive to light, he cannot recall the incident or the
40. game around it, and is not oriented to time or place.
41. A 14-year-old boy presents via ambulance 1 h following a collision
42. with another player where they collided heads during a rugby
43. game. He was knocked out for 2 min. There was no reported
44. seizure activity. Currently he is complaining of a headache and
45. nausea. Medical history reveals asthma and a concussion 2 years
46. prior, medications = ventolin p.r.n. On examination he has a GCS
47. of 13 (Eye 3, Movement 6, Verbal 4), no signs of basal skull
48. fracture, hasa2cmtender haematoma on his forehead, pupils are
49. equal and reactive to light, he cannot recall the incident or the
50. game around it, and is not oriented to time or place.

Below is a clinical vignette for a patient that presents to your emergency department. Please read the vignette and answer the following questions.

A 14-year-old boy presents via ambulance 1 h following a collision with another player where they collided heads during a rugby game. He was knocked out for 2 min. There was no reported seizure activity. Currently he is complaining of a headache and nausea. Medical history reveals asthma and a concussion 2 years prior, medications = ventolin p.r.n. On examination he is moving all extremities purposefully, is speaking in clear sentences, but doesn’t remember the event. He has no signs of basal skull fracture, has a 2cm tender hematoma on his forehead, pupils are equal and reactive to light, he cannot recall the incident or the game around it, and is not oriented to time or place.

**2Q1.** Would you order diagnostic imaging immediately for this patient?

| 1 | O | Yes |
| --- | --- | --- |
| 0 | O | No |

**[SHOW 2Q2 IF 2Q1 = “Yes”]**

**2Q2.** What diagnostic imaging would you order for your immediate plan? (select any that apply)

|  |  | **Diagnostic Imaging Choice (select any that apply)**  ***[RANDOMIZE ITEMS BELOW]*** |  |
| --- | --- | --- | --- |
| 1 | □ | Skull X-ray |  |
| 2 | □ | CT scan |  |
| 3 | □ | MRI scan |  |
| 4 | □ | Other (please specify: ______) | ***[FREE TEXT BOX]*** |

**[SHOW 2Q3 IF 2Q1 = “No”]**

**2Q3.** What is your immediate plan for this patient? (select any that apply)

|  |  | **Management Choice (select any that apply)**  ***[RANDOMIZE ITEMS BELOW]*** |  |
| --- | --- | --- | --- |
| 1 | □ | Discharge home now |  |
| 2 | □ | Give analgesia |  |
| 4 | □ | Observation in ED / SOU / hospital for ___ hours: | ***[FREE TEXT BOX]*** |
| 5 | □ | Admit to hospital |  |
| 6 | □ | Refer for follow-up with outpatient provider |  |
| 7 | □ | Other (please specify: ______) | ***[FREE TEXT BOX]*** |

**[SHOW 2Q5 IF 2Q1 = “Yes”]**

**2Q5.** The results of the diagnostic imaging you order are unremarkable. What is your management plan now? (select any that apply)

|  |  | **Management Choice (select any that apply)**  **[RANDOMIZE OPTIONS]** |  |
| --- | --- | --- | --- |
| 1 | □ | Discharge home now |  |
| 2 | □ | Give analgesia |  |
| 3 | □ | Order more diagnostic imaging |  |
| 4 | □ | Observation in ED / SOU / hospital for ___ hours: | ***[FREE TEXT BOX]*** |
| 5 | □ | Admit to hospital |  |
| 6 | □ | Refer for follow-up with outpatient provider |  |
| 7 | □ | Other (please specify: ______) | ***[FREE TEXT BOX]*** |

# **Section 3. Motivation to improve**

For each of the following statements, please indicate how true it is for you using the following scale:

| **1** | **2** | **3** | **4** | **5** | **6** | **7** |
| --- | --- | --- | --- | --- | --- | --- |
| Not at all true |  |  | Somewhat true |  |  | Very true |

**3Q1. Value/Usefulness (internalization of motivation)**

|  |  | **1** | **2** | **3** | **4** | **5** | **6** | **7** |
| --- | --- | --- | --- | --- | --- | --- | --- | --- |
|  | **Statement *[RANDOMIZE ITEMS BELOW]*** | Not at all true |  |  | Somewhat true |  |  | Very true |
| 1 | I think that making quick disposition decisions is useful to improve patient outcomes. | O | O | O | O | O | O | O |
| 2 | I think it is important to make quick disposition decisions for patient satisfaction. | O | O | O | O | O | O | O |
| 3 | I think making quick disposition decisions could help me deliver better care. | O | O | O | O | O | O | O |
| 4 | I believe making quick disposition decisions could be beneficial to me. | O | O | O | O | O | O | O |
| 5 | I think making quick disposition decisions is important. | O | O | O | O | O | O | O |

**3Q2. Perceived Competence**

|  |  | **1** | **2** | **3** | **4** | **5** | **6** | **7** |
| --- | --- | --- | --- | --- | --- | --- | --- | --- |
|  | **Statement *[RANDOMIZE ITEMS BELOW]*** | Not at all true |  |  | Somewhat true |  |  | Very true |
| 1 | I think I am pretty good at making quick disposition decisions. | O | O | O | O | O | O | O |
| 2 | I think I do pretty well at making quick disposition decisions, compared to others. | O | O | O | O | O | O | O |
| 3 | I am satisfied with how quickly I make disposition decisions. | O | O | O | O | O | O | O |
| 4 | I feel pretty skilled in making quick disposition decisions | O | O | O | O | O | O | O |

**3Q3. Autonomy Support**

|  |  | **1** | **2** | **3** | **4** | **5** | **6** | **7** |
| --- | --- | --- | --- | --- | --- | --- | --- | --- |
|  | **Statement *[RANDOMIZE ITEMS BELOW]*** | Not at all true |  |  | Somewhat true |  |  | Very true |
| 1 | I feel that the ED medical directors provide me choices and options. | O | O | O | O | O | O | O |
| 2 | I feel understood by the ED medical directors. | O | O | O | O | O | O | O |
| 4 | The ED medical directors convey confidence in my ability to do well at my job. | O | O | O | O | O | O | O |
| 7 | The ED medical directors encourage me to ask questions. | O | O | O | O | O | O | O |
| 10 | The ED medical directors listen to how I would like to do things. | O | O | O | O | O | O | O |
| 14 | The ED medical directors try to understand how I see things before suggesting a new way to do things. | O | O | O | O | O | O | O |

# **Section 6. Response to Performance Feedback Tool & Technology Acceptance Model**

**6Q1. Usefulness**

Below are a few questions about the tool we showed you earlier that provides performance feedback for emergency medicine physicians. You may review the prototype at the following links.

Summary Page: **[INCLUDE LINK TO TOP-LINE SUMMARY PAGE OF PERFORMANCE FEEDBACK TOOL]**

Detailed Page: **[INCLUDE LINK TO DRILL-DOWN PAGE OF PERFORMANCE FEEDBACK TOOL]**

Rate your level of agreement for the following statements using the scale below.

| **1** | **2** | **3** | **4** | **5** | **6** | **7** |
| --- | --- | --- | --- | --- | --- | --- |
| Disagree strongly |  |  | Neutral |  |  | Agree strongly |

|  |  | **1** | **2** | **3** | **4** | **5** | **6** | **7** | **Do you have any comments or feedback about your selected rating?** |
| --- | --- | --- | --- | --- | --- | --- | --- | --- | --- |
|  |  | Disagree strongly |  |  | Neutral |  |  | Agree Strongly |  |
| 1 | This performance feedback tool would enable me to accomplish tasks more quickly. | O | O | O | O | O | O | O | ***[FREE TEXT BOX]*** |
| 2 | This performance feedback tool would improve my job performance. | O | O | O | O | O | O | O | ***[FREE TEXT BOX]*** |
| 3 | This performance feedback tool would increase my productivity. | O | O | O | O | O | O | O | ***[FREE TEXT BOX]*** |
| 4 | This performance feedback tool would enhance my effectiveness on the job. | O | O | O | O | O | O | O | ***[FREE TEXT BOX]*** |
| 5 | This performance feedback tool would make it easier to do my job. | O | O | O | O | O | O | O | ***[FREE TEXT BOX]*** |
| 6 | I would find this performance feedback tool useful in my job. | O | O | O | O | O | O | O | ***[FREE TEXT BOX]*** |

**6Q2. Ease of use**

You may review the prototype of the performance feedback tool shown earlier at the following links.

Summary Page: **[INCLUDE LINK TO TOP-LINE SUMMARY PAGE OF PERFORMANCE SUMMARY TOOL]**

Detailed Page: **[INCLUDE LINK TO DRILL-DOWN PAGE OF PERFORMANCE SUMMARY TOOL]**

Rate your level of agreement with the following statements.

|  |  | **1** | **2** | **3** | **4** | **5** | **6** | **7** | **Do you have any comments or feedback about your selected rating?** |
| --- | --- | --- | --- | --- | --- | --- | --- | --- | --- |
|  |  | Disagree strongly |  |  | Neutral |  |  | Agree Strongly |  |
| 1 | I believe that learning to use the performance feedback tool would be easy for me. | O | O | O | O | O | O | O | ***[FREE TEXT BOX]*** |
| 2 | I would find it easy to do what I want it to do using the performance feedback tool | O | O | O | O | O | O | O | ***[FREE TEXT BOX]*** |
| 3 | I expect that my interaction with the performance feedback tool would be clear and understandable. | O | O | O | O | O | O | O | ***[FREE TEXT BOX]*** |
| 4 | I believe that the performance feedback tool is flexible to interact with. | O | O | O | O | O | O | O | ***[FREE TEXT BOX]*** |
| 5 | It would be easy for me to become skillful at using the performance feedback tool. | O | O | O | O | O | O | O | ***[FREE TEXT BOX]*** |
| 6 | In my daily work, I would find the performance feedback tool easy to use. | O | O | O | O | O | O | O | ***[FREE TEXT BOX]*** |

**6Q3. Relevance of Metrics**

You may review the prototype of the performance feedback tool shown earlier at the following links.

Summary Page: **[INCLUDE LINK TO TOP-LINE SUMMARY PAGE OF PERFORMANCE SUMMARY TOOL]**

Detailed Page: **[INCLUDE LINK TO DRILL-DOWN PAGE OF PERFORMANCE SUMMARY TOOL]**

Rate your level of agreement with the following statements.

|  |  | **1** | **2** | **3** | **4** | **5** | **6** | **7** |
| --- | --- | --- | --- | --- | --- | --- | --- | --- |
|  |  | Disagree strongly |  |  | Neutral |  |  | Agree Strongly |
| 1 | Knowing the overall length of stay of my patients is important to me. | O | O | O | O | O | O | O |
| 2 | Knowing my time to disposition decision is important to me. | O | O | O | O | O | O | O |
| 3 | Knowing how many tests I order is important to me. | O | O | O | O | O | O | O |
| 9 | Overall, the metrics on this performance feedback tool are important to me. | O | O | O | O | O | O | O |

**6Q4. Belief in ability to affect metrics**

You may review the prototype of the performance feedback tool shown earlier at the following links.

Summary Page: **[INCLUDE LINK TO TOP-LINE SUMMARY PAGE OF PERFORMANCE SUMMARY TOOL]**

Detailed Page: **[INCLUDE LINK TO DRILL-DOWN PAGE OF PERFORMANCE SUMMARY TOOL]**

Rate your level of agreement with the following statements.

|  |  | **1** | **2** | **3** | **4** | **5** | **6** | **7** |
| --- | --- | --- | --- | --- | --- | --- | --- | --- |
|  |  | Disagree strongly |  |  | Neutral |  |  | Agree Strongly |
| 1 | I can affect the overall length of stay of my patients. | O | O | O | O | O | O | O |
| 2 | I can affect the overall time to disposition decision of my patients. | O | O | O | O | O | O | O |
| 3 | I can affect the number of tests I order. | O | O | O | O | O | O | O |
| 99 | Overall, I can affect the metrics on this performance feedback tool. | O | O | O | O | O | O | O |

**6Q5.** Please identify 3 to 5 words or short phrases which you feel best describe the performance feedback tool we’ve shown you. An example is provided below.

*Example: “User-friendly”*

| **[*FREE TEXT BOX]*** |
| --- |
| **[*FREE TEXT BOX]*** |
| **[*FREE TEXT BOX]*** |
| **[*FREE TEXT BOX]*** |
| **[*FREE TEXT BOX]*** |

**6Q6.** How likely are you to recommend this performance feedback tool to your colleagues and peers to visualize and improve their performance as ED physicians?

You may review the prototype of the performance feedback tool shown earlier at the following links.

Summary Page: **[INCLUDE LINK TO TOP-LINE SUMMARY PAGE OF PERFORMANCE SUMMARY TOOL]**

Detailed Page: **[INCLUDE LINK TO DRILL-DOWN PAGE OF PERFORMANCE SUMMARY TOOL]**

| **0** | **1** | **2** | **3** | **4** | **5** | **6** | **7** | **8** | **9** | **10** |
| --- | --- | --- | --- | --- | --- | --- | --- | --- | --- | --- |
| Not at all likely |  |  |  |  | Neutral |  |  |  |  | Extremely likely |
| O | O | O | O | O | O | O | O | O | O | O |

**6Q7.** **[ONLY SHOW IF 6Q11 < 9]** What would you improve in the performance feedback tool to increase your likelihood of recommending it to your colleagues and peers?

| **[*FREE TEXT BOX]*** |
| --- |

**Section 7. End**

**7Q1.** Do you have any other comments you would like to share with us? (skip if no)

| **[*FREE TEXT BOX]*** |
| --- |
