# Supplementary material for: Involving end-users in the design of an audit and feedback intervention in the emergency department setting – a mixed methods study
Source: BMC Health Serv Res. 2019 Apr 29;19:270. doi: 10.1186/s12913-019-4084-3 (PMC6489283; doi:10.1186/s12913-019-4084-3)
Supplement: Supplementary file 3 — Supplemental Tables and Figures. The supplemental tables and figures references throughout the paper. (DOCX 272 kb) [file 12913_2019_4084_MOESM3_ESM.docx]

**Table S1. Characteristics of interviewees.**

|  | **Initial interviews** | **Iterative design interviews** |
| --- | --- | --- |
| n | 8 | 5* |
| Gender   - Female - Male | 4 (50)  4 (50) | 3 (60)  2 (40) |
| Primary role (n, %)   - Academic - Clinical - Leadership | 3 (37.5)  3 (37.5)  2 (25) | 3 (60)  2 (40)  0 (0) |
| Employer (n, %)   - USC - LA County Department of Health | 6 (75)  2 (25) | 5 (100)  0 (0) |
| Years since start residency (median, range) | 12 (9-45) | 13 (9-21) |
| Years at LAC+USC ED (median, range) | 8 (2-31) | 9 (4-21) |
| Duration interview in minutes (median, range) | 57 (35-77) | 36 (33-56) |

*Legend: *4 interviewees were included in both the initial round of interviews and the iterative design interviews. Abbreviations: ED = emergency department; n = number; LA = Los Angeles; LAC+USC = Los Angeles County + University of Southern California; USC = University of Southern California.*

**Table S2. Measures discussed in interviews.**

| **Measure type** | **Metric** |
| --- | --- |
| Summary data | Number of patients, discharge disposition (% admitted to hospital, discharged, or admitted for observation), left without being seen, left before treatment complete, left against medical advice. |
| LOS measures | Total LOS, time to first provider, provider to disposition decision time, boarding time, time between test results and disposition decision. |
| Utilization data | N CT scans, MR scans, labs, repeat labs, consultations, narcotics. |
| Outcomes | Return rates, LOS in hospital after admission, deaths, patient satisfaction, complaints. |

**Table S3. Characteristics of survey respondents.**

|  | **Pre-test** | **Post-test** | **Pre- and post-test** |
| --- | --- | --- | --- |
| N | 42 | 21 | 19 |
| Involved in development – *n (%)* | 9 (21) | 5 (24) | 5 (26) |
| Male gender – *n (%)* | 23 (55) | 13 (62) | 12 (63) |
| Years experience *– median (IQR)* | 10 (6-15) | 10 (6-12) | 10 (7-14) |
| Years in this ED *– median (IQR)* | 6.5 (2-10) | 5 (2-8) | 6 (2-10) |

*Legend: ED = emergency department; IQR = interquartile range; n = number.*

**Figure S1. Monthly email report.**
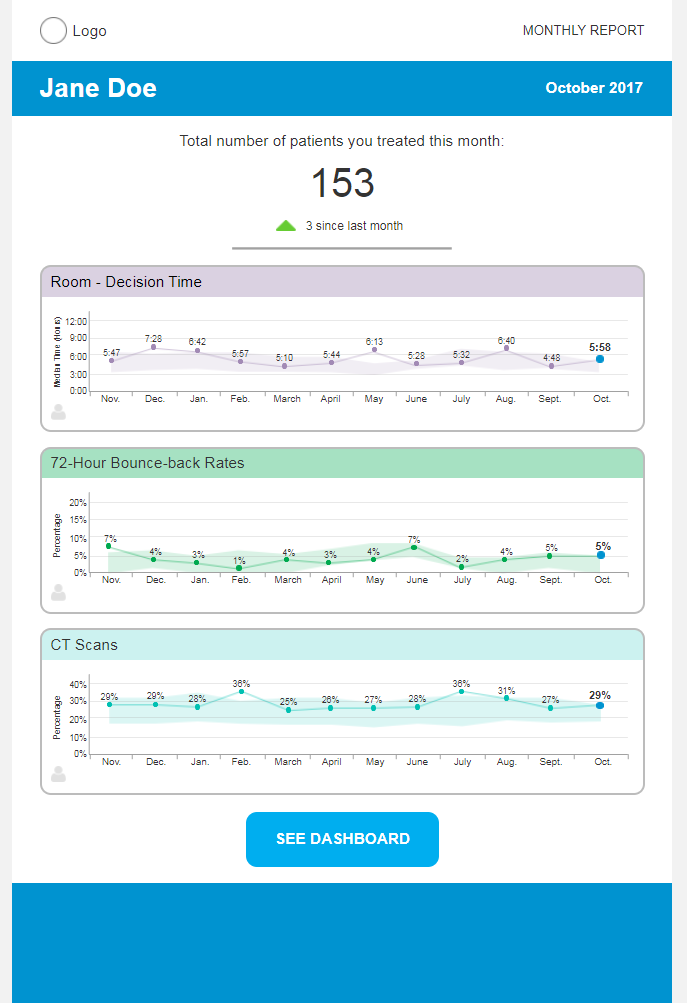

*Legend: Prototype monthly email report. The name Jane Doe is a false name.*

**Figure S2. Performance feedback dashboard.**
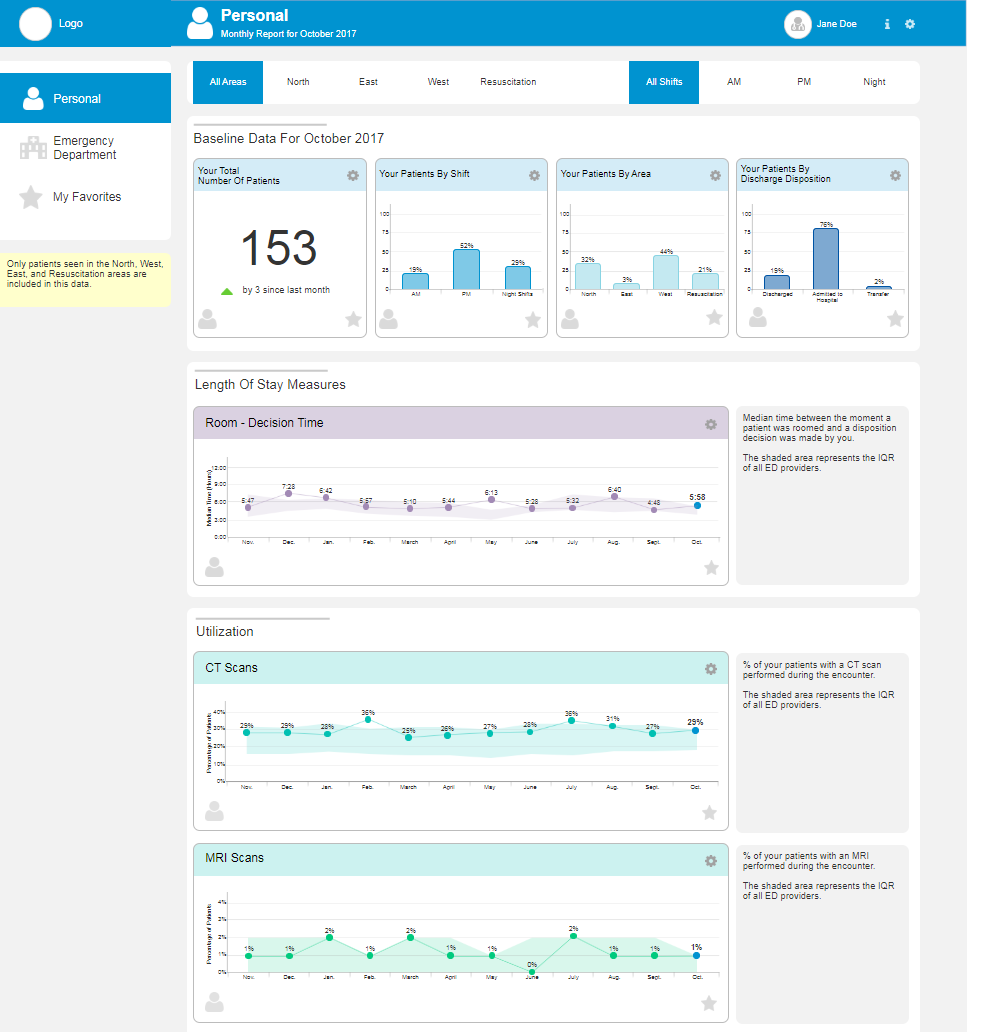

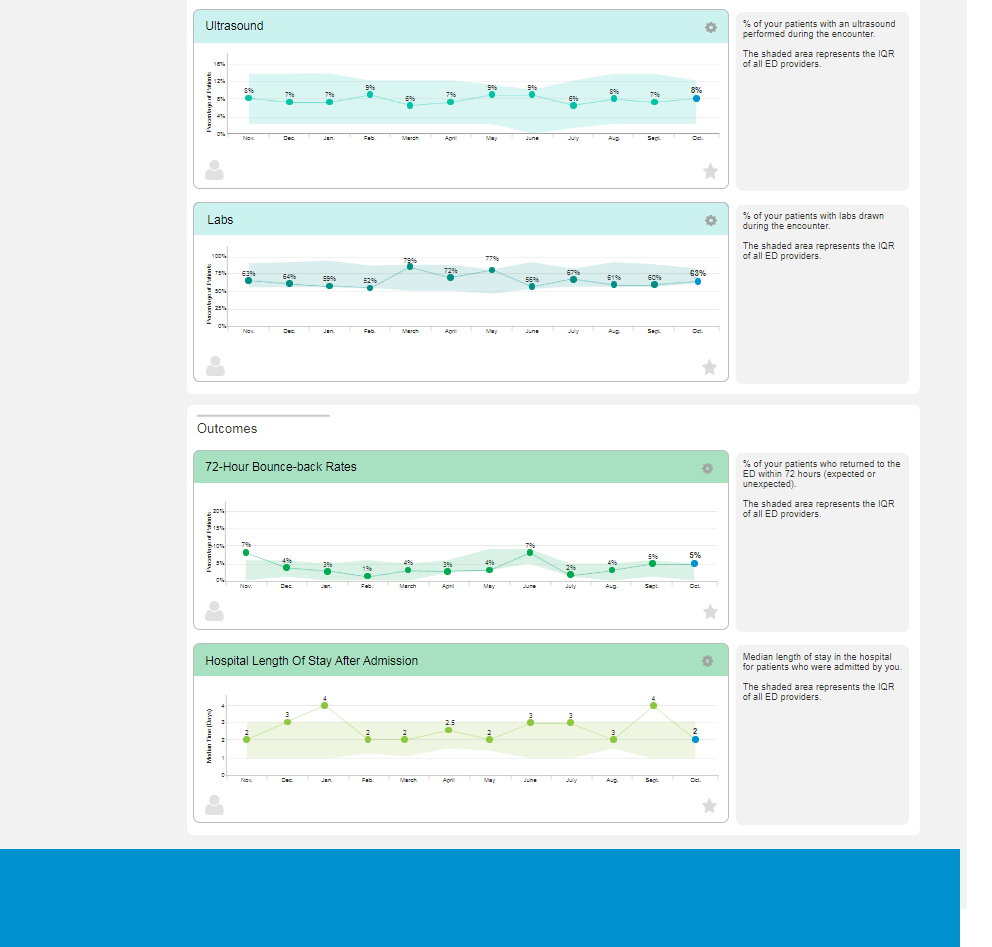


*Legend: Prototype performance feedback dashboard. The name Jane Doe is a false name.*
